# Ipsilateral elbow and shoulder dislocations in older people: Which joint should be reduced first?: A rare case report and literature review

**DOI:** 10.1097/MD.0000000000042080

**Published:** 2025-04-04

**Authors:** Mei-Ren Zhang, Jian-Hui Hu, Xiao Zeng, You-Cong Feng, Hai-Yun Chen

**Affiliations:** a Orthopedics Trauma Department Zhuhai Branch, Guangzhou University of Chinese Medicine Second Clinical Medicine College, Guangdong Province Hospital of Traditional Chinese Medicine Zhuhai Branch, Zhuhai City, PR China; b Guangzhou University of Chinese Medicine Second Clinical Medicine College, Guangzhou, PR China; c Orthopedics Trauma Department Zhuhai Branch, Guangzhou University of Chinese Medicine Second Affiliated Hospital, Guangdong Provincial Hospital of Chinese Medicine, Zhuhai City, PR China.

**Keywords:** close reduction, elbow dislocation, ipsilateral, shoulder dislocation

## Abstract

**Rationale:**

Ipsilateral simultaneous shoulder and elbow dislocations are rare and complex injuries. Only a few case reports have been published in the literature. Therefore, little is known about the mechanism of injury and its treatment.

**Patient concerns:**

An 83-year-old woman presented with severe pain in the right shoulder and elbow after a fall. Radiographs revealed an unusual ipsilateral dislocation of both the elbow and shoulder, without any fractures.

**Diagnoses:**

Physical examination and radiographs confirmed an unusual ipsilateral dislocation of both the elbow and shoulder, without any fractures.

**Interventions:**

The shoulder dislocation was successfully manipulated without any anesthesia by a doctor first. Following this, the elbow dislocation was successfully treated by the same doctor, with the help of 2 assistants. The patient was then immobilized in a posterior plaster slab for the elbow, and a triangular sling for the shoulder, for a total of 3 weeks.

**Outcomes:**

Seven months after the injury, almost full range of motion was achieved on both joints, with only mild pain, The shoulder score, assessed using the American Shoulder and Elbow Surgeons Scale, was 80, while the elbow score was 87 at the last follow-up.

**Lessons:**

Ipsilateral elbow and shoulder dislocations are very unusual injuries for older people. However, we should not overlook associated injuries on initial assessment, to avoid missing a diagnosis. Excellent functional outcomes may be achievable by an initial closed reduction of the shoulder, followed by treatment of the elbow, particularly in older patients without associated fractures.

## 1. Introduction

While isolated shoulder or elbow dislocations are frequently seen, simultaneous ipsilateral shoulder and elbow dislocations are rare. Only a few cases reports have been published in the literature. A search was made in PubMed database, which identified only 14 previously reported cases in the English literature.^[1–14]^ We present a case of an ipsilateral elbow and shoulder dislocation without associated fractures. As one might expect this combination was usually the result of high-energy forces, so such a combination of injuries has not previously been described for more than 60 years. The patient was an 83-year-old woman, and was the oldest known patient suffering from this rare injury in the previous reported literature. The objective of this clinical case is to increase the awareness that this rare injury may also present in older people in emergency departments.

## 2. Case presentation

**Figure 1.**
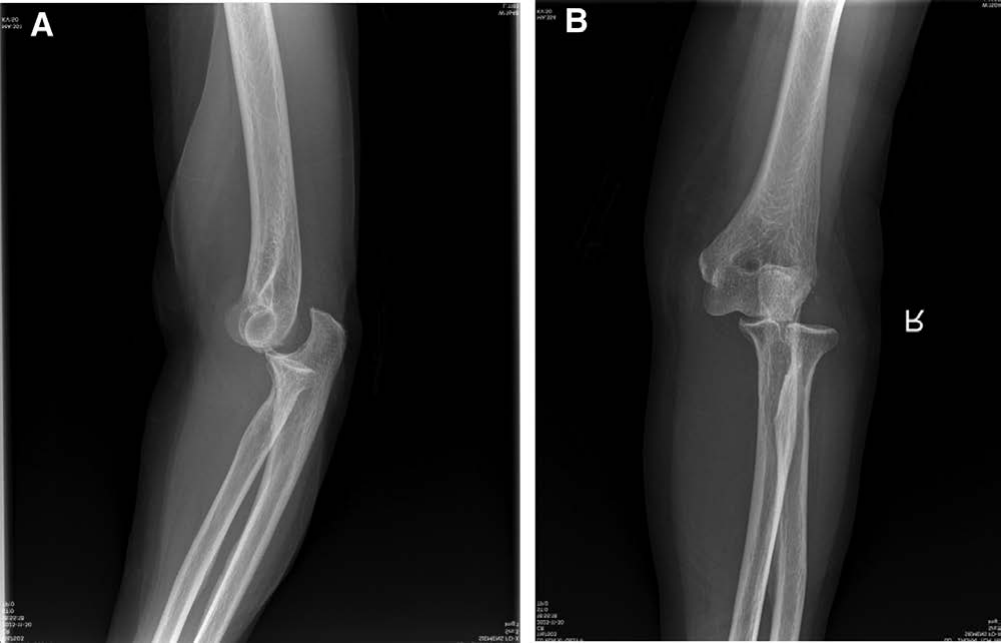
A–P view (A) and lateral view (B) radiographs of right elbow after injury showed an obviously migrated lateral dislocation of elbow without fracture.

**Figure 2.**
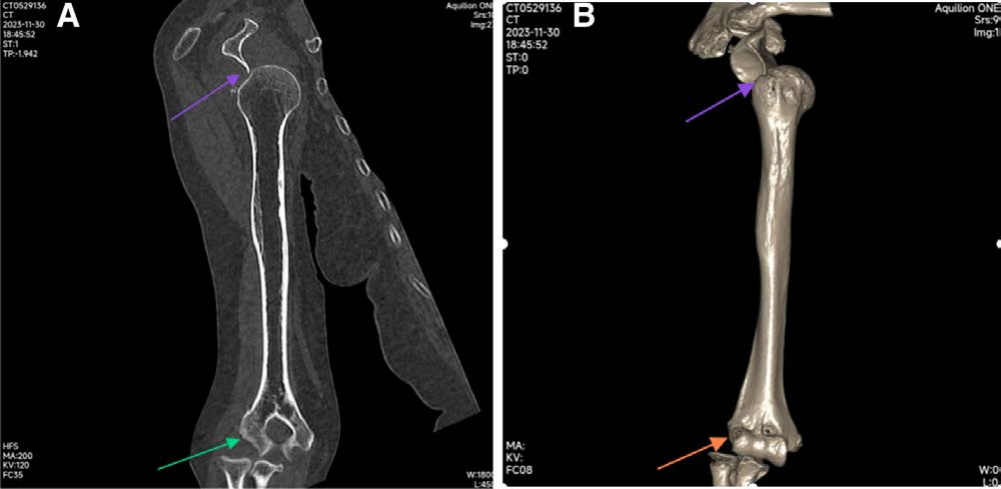
Coronal view (A) images show anterior subglenoid dislocation of shoulder (yellow arrow) with lateral dislocation of elbow (green arrow); 3D CT (B) also show ipsilateral anterior subglenoid dislocation (purple arrow) and lateral dislocation of elbow without fracture (red arrow).

**Figure 3.**
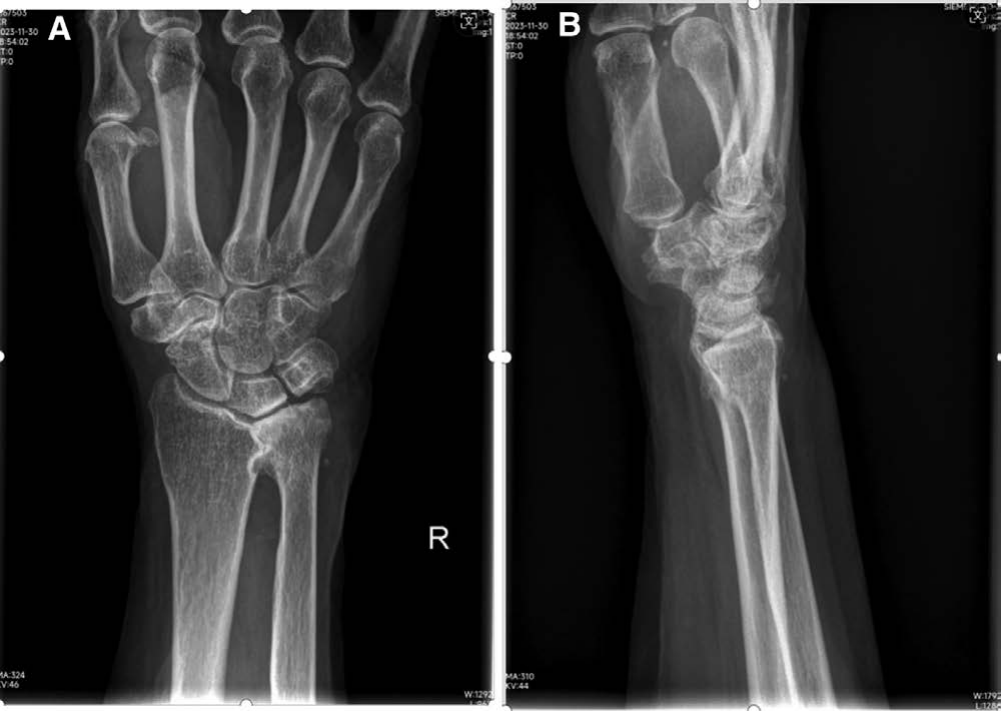
A–P view (A) and lateral view (B) radiographs of right wrist after injury showed no injury or dislocation of the inferior radioulnar joint.

**Figure 4.**
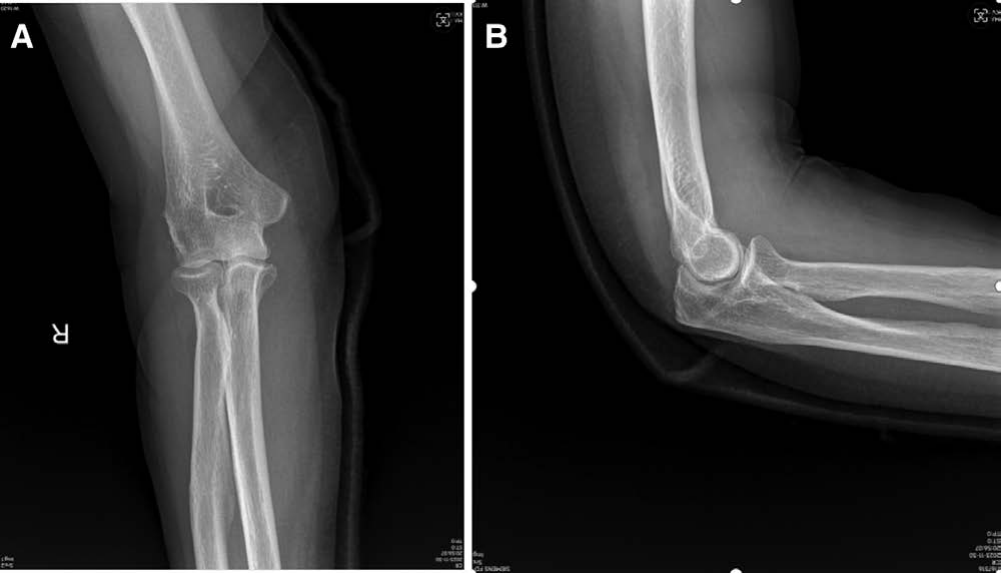
A–P view (A) and lateral view (B) radiographs of right elbow after reduction showed normal elbow joint with corrected of lateral dislocation.

**Figure 5.**
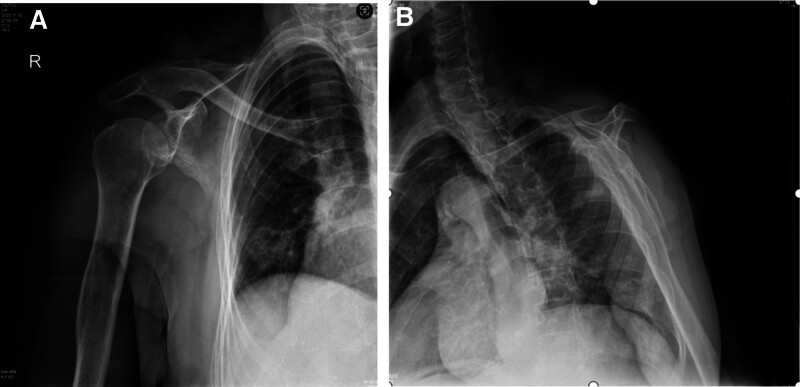
A–P view (A) and Y view (B) radiographs of shoulder joint view after reduction showed normal shoulder joint with corrected of anterior subglenoid dislocation.

**Figure 6.**
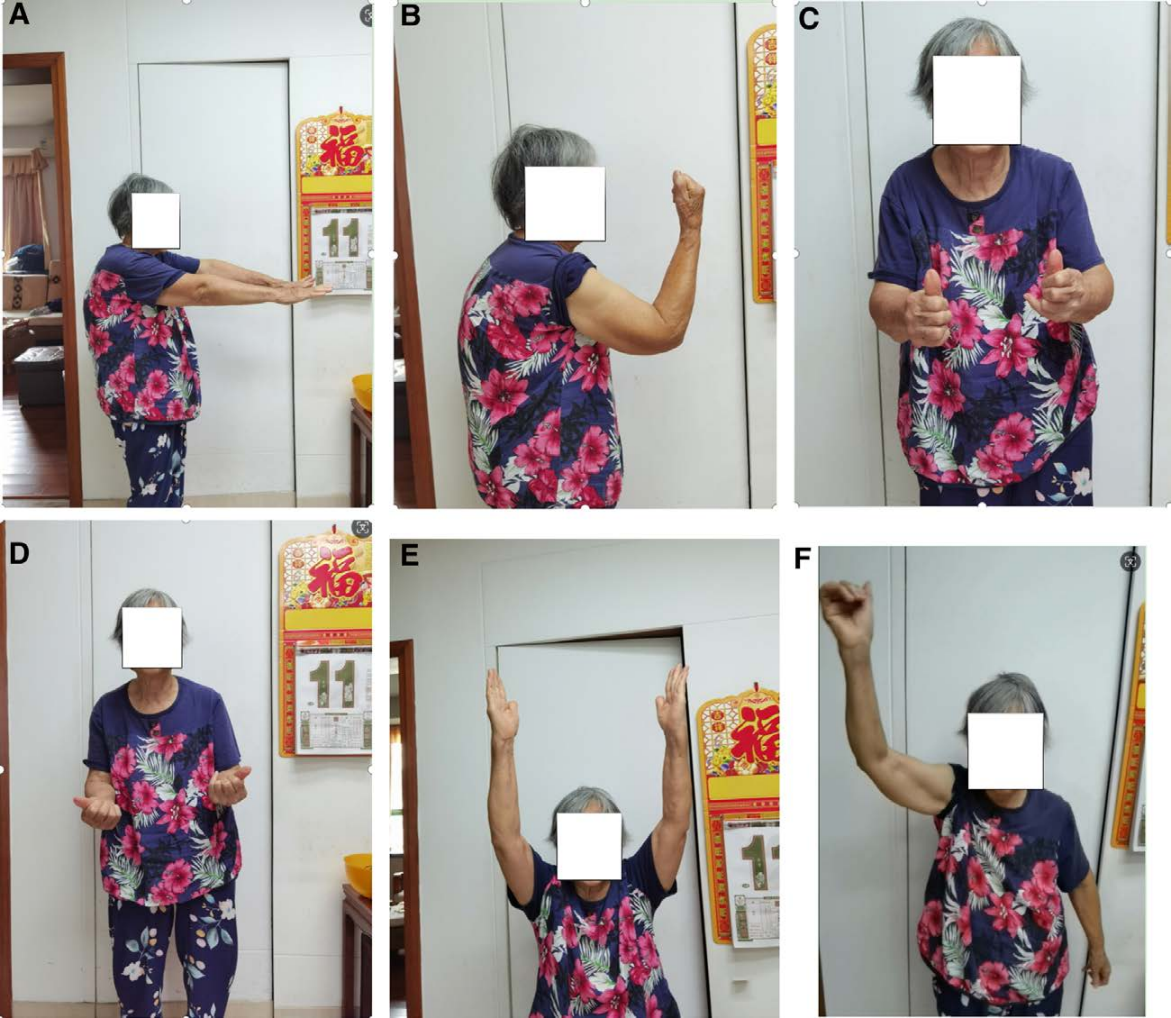
Seven months after injury showed good function of elbow (A–D) and shoulder joint (E, F).

An 83-year-old female patient presented to our hospital emergency department with right elbow and shoulder pain following a fall due to sudden dizziness. The patient denied epileptic episodes before injury, had stable vital signs, and showed no altered consciousness. Deformity and pain of the right elbow and shoulder, with accompanying limitation in range of motion (ROM), were found in her initial evaluation. Fortunately her brachial, radial, and ulnar arteries were palpable, without any motor deficit. She had a history of atrial fibrillation and had been taking medication to control it. She denied other medical history such as hypertension and diabetes as well as any history of surgery. Before the injury, he had good function joints of extremities and was self-reliant in daily living. Radiographic evaluation revealed several upper limb injuries, including a lateral elbow dislocation (Fig. 1A and B), anterior subglenoid dislocation of the shoulder (Fig. 2A and B), and fortunately, there was no injury or dislocation of the inferior radioulnar joint (Fig. 3A and B). Firstly, the shoulder dislocation was reduced by an experienced orthopedic trauma surgeon without anesthesia after admission, using the Kocher technique maneuvers without any difficulty while patient sitting in a back chair. Then, the elbow dislocations were treated by the same maneuvers. With the help of 2 assistants, the traction–countertraction technique was used. First, one of assistant applied countertraction at the distal humerus to keep the shoulder in adduction and internal rotation, while another assistant applied longitudinal traction on the forearm in a supinated position. Maneuvers manipulated the olecranon process with the forearm assistant gradually applying elbow flexion. The reductions were successful at the first attempt. The elbow was immobilized in flexion (90°) in a neutral position using a posterior plaster slab, and the shoulder was supported by a triangular sling, for a total of 3 weeks. After reduction, the square shoulder deformity disappeared, and the pain in the shoulder subsided immediately. Radiographic evaluation performed after the closed reduction confirmed appropriate joint reduction of the elbow (Fig. 4A and B) and shoulder (Fig. 5A and B). And then wrist and hand exercises were encouraged while the elbow was in plaster for 3 weeks and the shoulder in a sling for 6 weeks. External shoulder rotation was prohibited during this time. Rotator cuff strengthening and ROM exercises were prescribed for 6 weeks, followed by 3 months of active ROM. After 6 months of rehabilitation, she had regained good function of the right elbow—with mild limitations of flexion and pronation—and excellent function of the right shoulder with almost full range of movement. Seven months after injury, during the follow-up visit, the patient expressed had an active ROM with a 15° deficit of elbow flexion (Fig. 6A–D), and mild residual pain of the shoulder, without limitation of movement (Fig. 6E and F). She resumed her daily life activities, to pre-injury level. The articular range was also symmetrical with her contralateral limb. The shoulder score, assessed using the American Shoulder and Elbow Surgeons Scale, was 80, while the elbow score was 87 at last follow-up.

## 3. Discussion

Shoulder dislocations are the most common joint dislocations, the majority of dislocations are anterior where <3% are posterior,^[15]^ and shoulder dislocations are being almost 5 times more frequent (24 per 100,000 person-years)^[16]^ than elbow dislocations (5.2 per 100,000 person-years).^[17]^ The occurrence of ipsilateral elbow and shoulder dislocations are a very unusual clinical situation. A literature review identified 15 previously described cases.^[1–14]^ Of these cases, only 6^[1,5,9,11,13]^ reported ipsilateral elbow and shoulder dislocations without associated fractures around both joints. All of the cases were young patients (age from 21 to 54 years), with none over 60 years of age. There are several reasons for this unusual injury to seldom occur in older people. First, the mechanism of this injury was thought to be falling on a flexed elbow, with the shoulder in abduction,^[4,11,18]^ which seldom occurs during daily activities. Second, these injuries are also associated with high-energy trauma, which leads to fracture–dislocations rather than isolated dislocations, both for elbows and shoulders. The injury mechanism in this patient was likely due to dizziness, which caused her to fall within the confined space of an elevator. Instinctively, she raised her right upper limb in an abducted and externally rotated position to protect herself, leading to contact with the elevator wall. The impact caused the body’s weight to shift forward, forcing the right shoulder joint into hyperextension and resulting in its dislocation.

The violence continues to elastically immobilize humerus, resulting in the elbow valgus and posterolateral rotation of the forearm which was still in an abducted and externally rotated position, thus causing the elbow dislocation. This is particularly the case for older people because of underlying osteoporosis. The patient in this case was the oldest reported case of a patient suffering from this rare injury in the known literature.

## 4. Which joint should we reduce first in this very unusual clinical situation?

In 14 previously described cases, all dislocations were successfully treated with closed reduction. Three authors^[1,2,12]^ did not describe the order of reduction. In 2 of the cases,^[6,7]^ patient with humeral shaft fractures had the shoulder joint reduced first due to an unstable elbow joint. In the other 9 patients^[3–5,8–14]^ elbow reductions preceded the shoulder reductions; however 5^[3,4,8,11,13]^ of the shoulder dislocations were not found until after the elbow joint reduction due to missed diagnoses. All of these cases did not follow basic physical examination principles at the beginning, which is to routinely examine the adjacent joints of an injured joint to avoid overlooking associated injuries. The reason for reducing the elbow first was in order to have stable distal part. Shoulder reduction after the elbow reduction is much easier and safer, as the distal extremity is stable while reducing the shoulder.^[1,3,4,13]^

In our case, there were several reasons for us to perform the shoulder reduction after the elbow reduction. First, this patient presented with more severe pain at the shoulder area than elbow at that time. Second, given her age, the shoulder dislocation was very easy to reduce due to her relaxed shoulder joint muscles. This is why her shoulder was reduced solely by an orthopedic surgeon without any difficulty, and the shoulder area pain relieved immediately. Furthermore, for an older woman with osteoporosis, it was possible that serious complications, such as humeral head fracture–dislocations, could be caused during elbow reduction, particularly with the help of inexperienced assistants performing traction while shoulder joint was still dislocated. It would then be difficult to deal with once this serious complication occurs, leading to serious consequences for the patient. Kocher technique was one of the most commonly used in anterior dislocation of shoulder.^[19–24]^ Since the reduction of the shoulder joint would not aggravate the dislocation of elbow, we applied Kocher technique to this patient.

It was very important to choose one of the reduction maneuvers that kept the shoulder in adduction and internal rotation while performing elbow reduction. Additionally, it was important to avoid maneuvers that can destabilize the shoulder with abduction and external rotation, thereby losing the initial reduction obtained, and potentially causing more damage.

## 5. Conclusions

Our case suggests that while ipsilateral elbow and shoulder dislocations are very unusual injuries for older people, we should not overlook associated injuries at the initial assessment to avoid missing a diagnosis. Excellent functional outcomes may be achievable for this injury via closed reduction, with the shoulder treated first, and followed by elbow reduction, particularly for older people without associated fractures.

## Author contributions

**Conceptualization:** Mei-Ren Zhang.

**Data curation:** Mei-Ren Zhang, Jian-Hui Hu, Xiao Zeng, You-Cong Feng.

**Formal analysis:** Xiao Zeng, You-Cong Feng.

**Funding acquisition:** Hai-Yun Chen.

**Investigation:** You-Cong Feng.

**Methodology:** Jian-Hui Hu.

**Project administration:** Hai-Yun Chen.

**Resources:** You-Cong Feng.

**Software:** Xiao Zeng.

**Supervision:** Hai-Yun Chen.

**Writing – original draft:** Mei-Ren Zhang.

**Writing – review & editing:** Mei-Ren Zhang.
